# Combining mitochondrial and nuclear genome analyses to dissect the effects of colonization, environment, and geography on population structure in *Pinus tabuliformis*

**DOI:** 10.1111/eva.12697

**Published:** 2018-09-24

**Authors:** Hanhan Xia, Baosheng Wang, Wei Zhao, Jin Pan, Jian‐Feng Mao, Xiao‐Ru Wang

**Affiliations:** ^1^ Advanced Innovation Center for Tree Breeding by Molecular Design National Engineering Laboratory for Tree Breeding College of Biological Sciences and Technology Beijing Forestry University Beijing China; ^2^ Key Laboratory of Plant Resources Conservation and Sustainable Utilization South China Botanical Garden Chinese Academy of Sciences Guangzhou China; ^3^ Department of Ecology and Environmental Science UPSC Umeå University Umeå Sweden

**Keywords:** demographic history, genotyping‐by‐sequencing, local adaptation, niche modeling, population structure

## Abstract

The phylogeographic histories of plants in East Asia are complex and shaped by both past large‐scale climatic oscillations and dramatic tectonic events. The impact of these historic events, as well as ecological adaptation, on the distribution of biodiversity remains to be elucidated. *Pinus tabuliformis* is the dominant coniferous tree in northern China, with a large distribution across wide environmental gradients. We examined genetic variation in this species using genotyping‐by‐sequencing and mitochondrial (mt) DNA markers. We found population structure on both nuclear and mt genomes with a geographic pattern that corresponds well with the landscape of northern China. To understand the contributions of environment, geography, and colonization history to the observed population structure, we performed ecological niche modeling and partitioned the among‐population genomic variance into isolation by environment (IBE), isolation by distance (IBD), and isolation by colonization (IBC). We used mtDNA, which is transmitted by seeds in pine, to reflect colonization. We found little impact of IBE, IBD, and IBC on variation in neutral SNPs, but significant impact of IBE on a group of outlier loci. The lack of IBC illustrates that the maternal history can be quickly eroded from the nuclear genome by high rates of gene flow. Our results suggest that genomic variation in *P. tabuliformis* is largely affected by neutral and stochastic processes, and the signature of local adaptation is visible only at robust outlier loci. This study enriches our understanding on the complex evolutionary forces that shape the distribution of genetic variation in plant taxa in northern China, and guides breeding, conservation, and reforestation programs for *P. tabuliformis*.

## 1 INTRODUCTION

Genetic differentiation results from both neutral and selective processes. Neutral demographic changes, like range shifts caused by large‐scale topographic movement and climatic oscillations, can result in increased population divergence via genetic drift. On the other hand, in situations where populations spread over heterogeneous landscapes, adaptation to distinct niches can also drive genetic divergence among local populations due to low fitness of immigrants or hybrids in their new environments, which limits the potential for gene flow (Nosil, Vines, & Funk, 2005). The consequences of these adaptive and neutral processes are not mutually exclusive, and natural populations are expected to experience them in combination (Orsini, Vanoverbeke, Swillen, Mergeay, & De Meester, 2013). An important step toward understanding the process of divergence is reliably establishing the demographic history of the species and the spatial distribution of its diversity, and determining how geography, demography, and environment affect the genetic structure of populations. Population demographic parameters such as the timing of population subdivision, effective population sizes, and the rate of gene flow between populations are important for identifying historic events (e.g., topographic changes and climatic oscillations) associated with population isolation/expansion and their consequences for the genetic composition of contemporary populations. Ecological niche modeling and multivariate genetic–environment association analysis can improve our ability to assess neutral and selective process that drive species subdivision and maintain genetic and morphological differentiation.

Northern China, as part of the “Sino‐Japanese Floristic Region,” has a complex topography characterized by a number of plains, basins, high mountains, and plateaus. The most recent profound tectonic events in north China were the uplift of the Qinling Mountains from the late Miocene and early Pliocene (7.28–3.60 million years ago, MYA) to the Pleistocene (Ge et al., 2012), and the latest uplift of the eastern Tibetan Plateau during the Pleistocene (Li, Fang, Pan, Zhao, & Song, 2001; but see Renner, 2016). The complex topography of high mountains and deep valleys in the Qinling Mountains and the eastern Tibetan Plateau have functioned as effective barriers to gene flow in many plant species (Chen, Du, Wang, & Wang, 2014; Jacques et al., 2014; Li, Liu et al., 2012; Lopez‐Pujol, Zhang, Sun, Ying, & Ge, 2011; Yuan, Cheng, & Zhou, 2012). By forming a huge physical obstacle for the northward movement of the monsoon, the Qinling Mountain Range also functions as an import climate boundary between northern and southern China (Rost, 1994), promoting niche divergence and adaptive differentiation among regional populations. In addition to the tectonic history, phylogeographic studies suggest that the Quaternary glaciation cycles from 2.58 MYA to 18,000 years ago had significant impacts on the genetic diversity of cold‐resistant forest species in northern China (Bai, Liao, & Zhang, 2010; Chen, Abbott, Milne, Tian, & Liu, 2008; Zeng, Wang, Liao, Wang, & Zhang, 2015). It is thus likely that past large‐scale climate fluctuations, dramatic geological movements, and ecological divergence have all affected the genetic diversity of plant taxa in this region. We predict that geographically separated and genetically differentiated populations of forest trees with long generation times have been and still are under the influence of both genetic drift and local adaptation.

*Pinus tabuliformis* is a major conifer forest species in northern and central China with an estimated distribution of about 630,000 km^2^ (Mao & Wang, 2011). Its range stretches almost 2,000 km from east to west and 1,200 km from north to south, and covers a broad‐ranging of environmental gradients (Wu, 1995; Ying, Chen, & Chang, 2004). Climate conditions, like growing season length, temperature, and precipitation, vary between the regions that are divided by the mountain chains. Pronounced phenotypic variations*,* including needle characteristics, seed weight, and growth traits, are recorded among geographic distant populations (Xu, 1992). Provenance trials show evidence of strong local adaptation as transplantation across climate zones results in high mortality and inferior growth of the transplanted populations (Xu, 1992; Zhao et al., 2014). As a major conifer species for reforestation in northern China, large breeding and tree improvement programs were initiated recently with the primary aim to increase forest coverage in harsh climate zones. Thus, a good understanding about the extent and nature of local adaptation and factors structuring genetic variation is important to guide forest management, conservation, and seed transfer for restoration and reforestation in *P. tabuliformis*.

Studies on the biogeography of *P. tabuliformis* using mitochondrial (mt) and chloroplast (cp) DNA markers revealed the structure of mt‐ and cpDNA variation across the species distribution and suggested that the species survived in multiple glacial refugia during the Last Glacial Maximum (LGM) and then colonized its current range during the postglacial period (Chen et al., 2008; Hao et al., 2018; Yang, Liu, Li, & Dyer, 2015). These studies provide valuable insights into the migration and colonization history of the species. However, genomewide pattern of genetic diversity and demographic history remains unclear for *P. tabuliformis*, and the impacts of selective processes on intraspecific differentiation have not been investigated to date. Cytoplasmic DNA markers are uniparentally inherited in pines (Wang, Szmidt, & Lu, 1996) and are valuable tools for tracing migration history. Organelle markers, on the other hand, may not capture the complete evolutionary history of a species due to their clonal transmission, small effective population size, and potential bias of genome capture. A whole‐genome approach, employing high‐throughput sequencing technology, will enable comparisons between more complex and potentially more precise demographic models and identification of gene loci involved in environmental adaptation. Restriction‐site‐associated DNA sequencing (e.g., genotyping‐by‐sequencing, GBS) is an effective approach for generating genetic data over thousands of loci and is increasingly being used to obtain population genomic perspective in non‐model forest trees (Parchman, Jahner, Uckele, Galland, & Eckert, 2018). However, to date, only a few investigations have explored GBS for dissecting the organization of genetic variation across landscapes in conifers (Johnson, Gaddis, Cairns, Konganti, & Krutovsky, 2017; Menon et al., 2018), probably due to difficulties in optimizing the technique in complex conifer genome analysis.

This study aims: (a) to gain a better view of genomewide patterns of diversity and population structure in *P. tabuliformis*; (b) to reconstruct the recent demographic history of this species; and (c) to evaluate the impact of environment, geography, and colonization history on intraspecific differentiation. To do this, we sampled populations across the range of *P. tabuliformis* and examined genetic variation in both nuclear and mt genomes. We used GBS to identify genomewide polymorphisms and simulate the demographic history of the species. We incorporated mtDNA information as a proxy for colonization history. MtDNA is maternally inherited and transmitted by seeds in pine (Wang et al., 1996), thus colonization history can persist in mtDNA for longer time due to low migration ability. We conducted ecological niche modeling to characterize the environmental variation across the species distribution as well. Finally, we partitioned the contribution of environment, geography, and colonization history to among‐population differentiation over neutral and candidate adaptive loci. Our integrated approach provides an example for dissecting complex evolutionary forces that shape population structure in conifer species, and improves our understanding of the divergence history of the northern China flora.

## 2 MATERIALS AND METHODS

### 2.1 Population sampling, DNA extraction, and mtDNA sequencing

**Figure 1:**
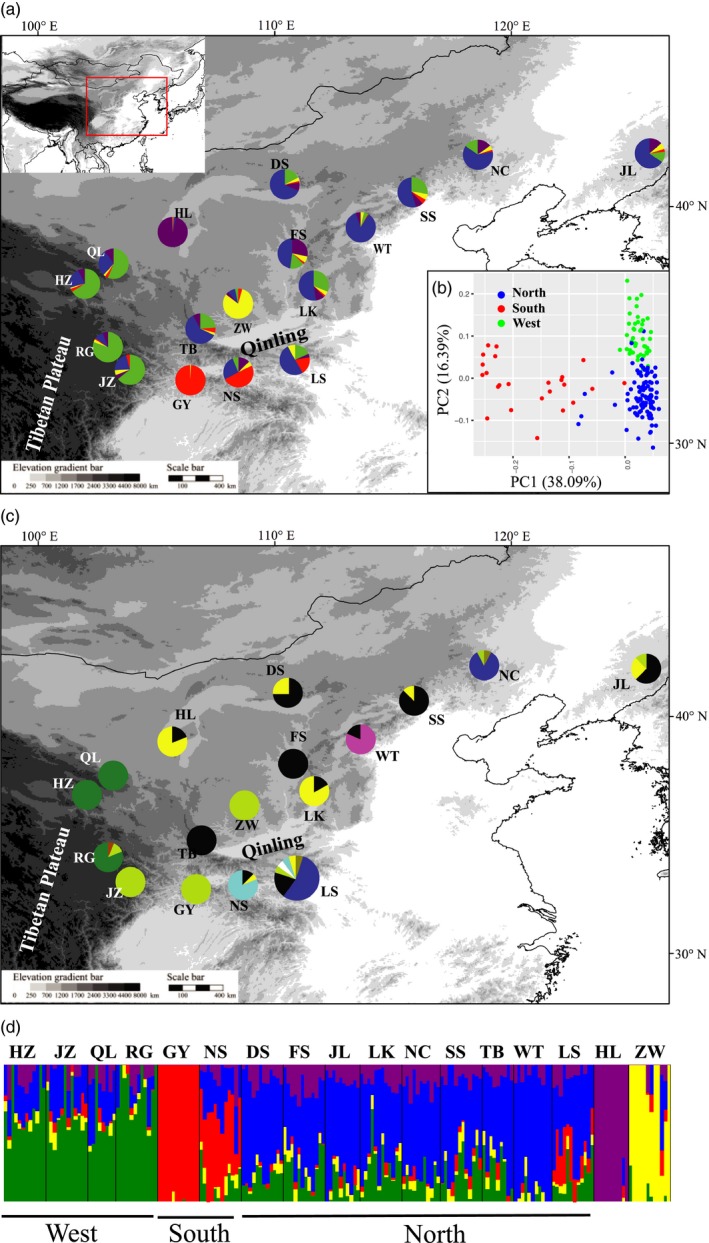
Population structure of the 17 populations of *P. tabuliformis* based on GBS (a, b and d) and mtDNA data (c). (a) Pie charts show the ancestry composition of each population for *K *=* *5 as inferred by Admixture. (b) Principal component analysis of 169 individuals from 15 populations of the three major groups (north, south, and west); the two single‐population groups, HL and ZW, were not included. (c) Pie charts show the proportions of mitotypes in each of the 17 populations. Population LS is enlarged to illustrate its mitotype composition more clearly. (d) Admixture assignment for 17 populations with *K *=* *5. Each bar represents an individual, with different colors corresponding to one of the ancestries

We sampled 17 populations throughout the range of *P. tabuliformis*. The distribution of these populations is illustrated in Figure 1. The name, location, and sample size of each population are listed in Table 1. Fourteen of the 17 populations have been characterized with respect to mtDNA variation previously (Wang, Mao, Gao, Zhao, & Wang, 2011). In this study, we included an additional three populations (FS, JZ, and LS) to match the GBS analyzed populations, and sequenced the three mtDNA segments (*nad*1 intron2, *nad*4 intron3, and *nad*5 intron1) as in Wang et al. (2011).

**Table 1:** Geographic locations, sample size (*N*), percentage of polymorphic loci (%*Poly*), the average heterozygosity per locus (*Het*), number of mitotypes (*n*_h_), and mitotype diversity (*H*_e_) of the 17 *Pinus tabuliformis* populations

Population	Longitude (E)	Latitude (N)	Altitude (m)	GBS			mtDNA		
				*N*	%*Poly*	*Het*	*N*	*n*_h_	*H*_e_
Jilin (JL)	126°36′	42°05′	500	10	85.5	0.229	8	3	0.607
Ningcheng (NC)	118°58′	42°16′	1300	11	85.0	0.217	13	3	0.295
Songshan (SS)	115°57′	40°27′	1700	12	89.6	0.228	8	2	0.250
Wutaishan (WT)	113°39′	38°53′	1328	11	81.8	0.219	16	2	0.325
Lingkongshan (LK)	112°02′	36°36′	1664	12	90.0	0.229	12	2	0.303
Fangshan (FS)	111°33′	37°56′	1500	12	87.7	0.214	8	1	0
Dongsheng (DS)	110°18′	40°47′	1400	12	87.9	0.221	8	2	0.429
Taibaishan (TB)	107°10′	34°02′	1200	9	81.9	0.222	8	1	0
Lushi (LS)	110°49′	33°44′	1878	12	91.2	0.235	20	6	0.679
North group				101	99.9	0.224	101	8	0.799
Jiuzhaigou (JZ)	103°47′	33°17′	2353	12	87.5	0.224	19	1	0
Qilianshan (QL)	103°26′	37°26′	2400	8	78.8	0.222	8	1	0
Ruoergai (RG)	103°21′	33°42′	1348	12	85.0	0.229	16	3	0.342
Huzhu (HZ)	102°27′	36°57′	2300	12	87.0	0.217	16	1	0
West group				44	98.9	0.223	59	3	0.488
Guangyuan (GY)	106°06′	32°37′	2947	12	85.8	0.231	16	1	0
Ningshan (NS)	108°23′	33°28′	1423	12	89.4	0.241	16	3	0.342
South group				24	95.9	0.236	32	4	0.599
Helanshan (HL)	105°55′	38°44′	2147	10	79.6	0.223	16	2	0.325
Ziwulin (ZW)	108°43′	35°38′	1118	12	79.4	0.217	8	1	0
Total				191		0.225	216	10	0.838

For each population, cones and/or needles were collected from 8 to 20 randomly selected matured trees that were at least 100 m apart. Genomic DNA was extracted from needles and germinating seedlings using a Plant Genomic DNA Kit (Tiangen, Beijing, China). For genotyping of mt genomes, we used DNA extracted from needles. For GBS, we extracted DNA from seedlings for 12 populations. One seed from each tree was germinated on Petri dishes, and genomic DNA was extracted from seedlings one week after germination. For the other five populations (HL, RG, TB, WT, and ZW), GBS was based on DNA from needles.

### 2.2 Genotyping‐by‐sequencing (GBS)

A GBS library was prepared for 192 individuals using a *Pst*I high fidelity restriction enzyme (New England Biolabs, MA, USA) following the protocol of Pan et al. (2015) with minor modification. Briefly, restriction enzyme digestion and adapter (with individual barcode) ligation were carried out simultaneously on 200 ng DNA from each sample. The digested and ligated DNA was then pooled, purified, and PCR amplified. The PCR products were separated on 1.8% high‐resolution agarose gels (Sigma, St. Louis, MO, USA), and fragments within the size range 400–500 bp (including 125–132 bp adaptors) were recovered. Paired‐end sequencing (2 × 125 bp) was performed on an Illumina HiSeq2500.

Adapter sequences and low‐quality bases (base quality <20) from the tail of each read were removed using Trimmomatic (Bolger, Lohse, & Usadel, 2014). The cleaned paired reads were cataloged using the process_radtags module of Stacks v1.46 with disable_rad_check parameter (Catchen, Amores, Hohenlohe, Cresko, & Postlethwait, 2011); reads shorter than 41 bases were discarded. We further removed the first five bases (enzyme recognition sites for *Pst*I) from each read using fastx_trimmer command in FASTX‐Toolkit (http://hannonlab.cshl.edu/fastx_toolkit/index.html). Then, all paired and orphan reads were mapped to the *Pinus taeda* draft genome v1.01 (Neale et al., 2014) with default parameters using BWA‐MEM algorithm (Li, 2013). Variants were called using SAMtools and BCFtools pipeline (Li, 2011). SNPs located in repetitive regions (reference to *P. taeda* genome v1.01), close (<5 bp) to indels and with mapping quality (MQ) <30, were discarded. Genotypes with genotype quality (GQ) <20 and sequencing depth <5 were masked as missing. SNPs with minor allele frequency (MAF) <0.05, proportion of missing genotypes (missing rate) ≥0.3, heterozygosity >50%, mean depth ≥400 (i.e., four times of the average depth of all SNPs), and genotyped in fewer than four individuals in any population were further filtered out. This final set of SNPs (4077) was used for all analyses in this study except for the demographic simulations, which was based on all SNPs including low‐frequency ones (see section *Inference of demographic history* for details).

### 2.3 Population genetic analyses

The population structure and admixture were investigated using Admixture v1.23 (Alexander, Novembre, & Lange, 2009). We run Admixture with *K *=* *1–17 and repeated the process 20 times with random seeds. A 10‐fold cross‐validation procedure was used for evaluating the runs with different *K* values in Admixture. We also applied a Δ*K* method (Evanno, Regnaut, & Goudet, 2005) to identify the best *K* value in the sampled populations, that is, the number of putative genetic groups. Because linkage between markers could inflate structure, we pruned the dataset by randomly pick up one SNP per GBS fragment and run Admixture on datasets including all SNPs (4077) and pruned SNPs (1679). Distribution of genetic variance was also assessed by principal component analysis (PCA) in EIGENSOFT v6.0 (Patterson, Price, & Reich, 2006). The percentage of polymorphic loci and per locus heterozygosity for each population and groups of populations, and population differentiation (Weir & Cockerham, 1984) at each SNP were calculated using VCFtools v1.012 (Danecek et al., 2011).

### 2.4 Inference of demographic history

Structure analysis of the GBS data identified three major groups in our samples, south, west, and north (see *Results* for details). We inferred the divergence history between these three groups by using a coalescent simulation‐based method implemented in fastsimcoal2 v2.6 (Excoffier, Dupanloup, Huerta‐Sanchez, Sousa, & Foll, 2013). We investigated three scenarios with different orders of branching: The south group diverged first, the north group diverged first, and the west group diverged first, respectively (Supporting Information Figure S1). For each scenario of divergence, we tested three models with different assumptions of population size changes in descendent populations including constant population sizes, exponential population growth, and recent exponential growth after the splitting of the groups (Supporting Information Figure S1, Table S1). In total, nine isolation‐with‐migration (IM) models were tested. Because missing data can lead to biased estimates of site frequency spectrum (SFS), we performed a down‐sampling procedure following Thome and Carstens (2016) to maximize the number of segregating sites retained for SFS estimation. To minimize biases when determining the ancestral allelic states, we generated folded SFS using Arlequin v.3.5 (Excoffier & Lischer, 2010). The final folded SFS contained 17,945 SNPs including those with low‐frequency alleles, that is, without filtering out SNPs with MAF <0.05. Considering the low confidence of rare alleles, we also ran fastsimcoal based on SFS without singleton (11,732 SNPs).

For each model, we performed 50 independent runs with 100,000 coalescent simulations as well as 10–40 conditional maximization algorithm cycles to estimate the global maximum‐likelihood parameters of each model. The best‐fitting demographic model was chosen based on the Akaike's weight of evidence following Excoffier et al. (2013). The goodness of fit of the best model was assessed by comparing the observed SFS with expected SFS, which was obtained by 10^5^ coalescent simulations under the maximum‐likelihood estimates of population parameters. The 95% confidence interval (CI) of the population parameters of the best model was calculated using a non‐parametric block bootstrap method, in which each GBS fragment is treated as an independent genomic block, resampled with replacement to generate 100 datasets. Generation time of 50 years and a mutation rate per year of 7 × 10^−10^ for the genus *Pinus* (Willyard, Syring, Gernandt, Liston, & Cronn, 2007) were used to convert model parameters to absolute values.

### 2.5 Outlier detection

We used two methods to identify loci that showed signatures of local adaptation: an allele frequency‐based method Pcadapt (Luu, Bazin, & Blum, 2017) and a genetic–environmental association method implemented in Bayenv2 (Günther & Coop, 2013). In Pcadapt, population structure is ascertained with PCA, and outliers are detected with respect to how they are related to population structure. Cattell's graphical rule was used to choose the number of principal components (*K*) to identify SNPs involved in local adaptation. Outliers were selected by performing the *q*‐value procedure at false discovery rate (FDR) 0.05 using the R package qvalue (Storey, Bass, Dabney, & Robinson, 2015).

Bayenv2 uses a set of putatively neutral loci to estimate the empirical pattern of covariance in allele frequencies between populations. It then uses this as a null model for testing correlation between individual SNPs and environmental variables (Günther & Coop, 2013). We generated a set of putatively neutral and independent SNPs (2156) by excluding significant loci identified by Pcadapt and LD pruning using indep‐pairwise (50 5 0.05) in PLINK (https://www.cog-genomics.org/plink/1.9/). This set of SNPs was then used to estimate the covariance matrix with 100,000 iterations. Covariance matrices were compared after three independent runs with different seed numbers to ensure convergence. Environmental correlations were assessed by averaging five independent runs of Bayenv2 with 100,000 Markov chain Monte Carlo (MCMC) iterations with different random seeds. We only considered the SNPs that were in the top 1% of the Bayes factor (BF) values, with BF >3, and in the top 5% of the absolute values of Spearman rank correlation coefficients (*ρ*) as robust candidate adaptive loci. Fourteen environmental variables with correlation coefficients <0.80 across the range of *P. tabuliformis* were used in Bayenv2 analyses (Supporting Information Table S2, see *Species distribution and ecological niche modeling* for details).

### 2.6 Redundancy analysis

A series of redundancy analyses (RDA) were conducted to estimate the degree to which genomic variation among *P. tabuliformis* populations can be explained by environment and demography. RDA involves a multiple linear regression followed by a PCA on the matrix of regression‐fitted values. We performed RDA in the R package VEGAN (Oksanen et al., 2016) using modified scripts from Nadeau, Meirmans, Aitken, Ritland, and Isabel (2016). We included a dependent matrix (allele frequencies for each population) and three independent matrices of climate variables (representing isolation by environment, IBE), geographic variables (representing isolation by distance, IBD), and mtDNA variables (representing isolation by colonization from glacial refugia, IBC). For the environment matrix, we ran a PCA on the 14 selected environmental factors (Supporting Information Table S2, see *Species distribution and ecological niche modeling*) using ade4 (Dray & Dufour, 2007) to reduce redundancy among variables, and retained PC1 and PC2 that jointly explained 76.2% of the environmental variation. For the geography matrix, we used a trend surface analysis to calculate second‐order polynomials and combinations of the coordinates of sampling locations (*x*,* y*,* xy*,* x* ^2^, *y* ^2^) to ensure that linear gradients in the data, as well as more complex patterns, were extracted. We further performed a forward selection with a stringent *α* value of 0.05 on geography to prevent overfitting of dependent and independent matrices. This resulted in the retention of one spatial variable (*y*). Results were similar if we included both geographic factors *x* and *y* (data not shown). The geographic factor matrix was centered, and the other three matrices were left untransformed. For the mtDNA matrix, after removing two singleton mitotypes, we first estimated the frequencies of each mitotype in each population and then performed PCA on the mitotype frequency matrix. PCs 1–4, which cumulatively explained 75.3% of the variations in mtDNA, were retained for RDA. Among‐population variation was partitioned into pure effects of environment, geography, and colonization history, as well as their combined effects. The significance of the partitioning was tested using the *anova.cca* function in VEGAN with 999 permutations. We performed RDA on datasets including neutral SNPs and outlier SNPs identified by the two outlier detection methods.

### 2.7 Species distribution and ecological niche modeling

Population genetic analysis identified three major groups over the distribution: south, north, and west (see *Results*). To understand whether these regions are ecologically differentiated, we retrieved species occurrence information from Mao and Wang (2011) and the Chinese Virtual Herbarium database (CVH; http://www.cvh.org.cn/englishindex.asp). A total of 164 unique occurrence records (Supporting Information Table S3) from the three geographic regions were used for ecological niche modeling. We screened 18 environmental variables used in a previous study of niche divergence between *P. tabuliformis* and closely related species (Mao & Wang, 2011) and retained 14 of them that had pairwise Spearman correlation coefficients <0.80 (Supporting Information Table S2) across the 164 occurrence sites. All selected environmental layers were converted to the same resolution at a grid cell size of 30” × 30” arc‐seconds (1 km^2^).

We simulated species distribution models (SDMs) via maximum entropy in Maxent 3.3.1 with the default settings (Phillips, Anderson, & Schapire, 2006). The predictive power of each model for the region where it was calibrated was evaluated with 25% of the occurrence dataset chosen at random and compared with the model output created using the remaining 75% of the dataset. Ten thousand background points were sampled to construct a predicted range of distribution for each *P. tabuliformis* group. Model accuracy was evaluated by assessing the area under the curve (AUC) of the receiver‐operating characteristic (ROC) plot. According to Swets’ scale, predictions are considered poor when AUC values are in the range 0.5–0.7, useful in the range 0.7–0.9, and good when greater than 0.9 (1 is perfect). Overlaying of grids, grid cell counting, and visualization of the model were conducted in ArcGIS 9.2 (Environmental Systems Research Institute, Redlands, CA). A SDM‐based background test was performed to examine whether each pair of the ecotypic groups was more or less divergent than would be expected from the differences in the local environmental backgrounds of the regions where they occur. This method compares the niche of a focal population with a set of pseudoniches modeled from random sampling of the geographic range of the sister population (Warren, Glor, & Turelli, 2008). Each test was performed for a pair of groups, in reciprocal directions. Background selection was achieved using the raster package in R and ArcGIS, and background tests were performed using ENMTools (Warren, Glor, & Turelli, 2010) with 100 random samplings.

We applied a niche space‐based multivariate test to assess the possibility that the allopatrically distributed ecotypic groups occupy similar niches. This test compares background divergence (*d*_b_) with observed niche divergence (*d*_n_) on the PCA‐reduced axes, with the null hypothesis *d*_b_ = *d*_n_ (McCormack, Zellmer, & Knowles, 2010). Niche divergence is supported if *d*_b_ < *d*_n_ and the observed niche divergence itself (*d*_n_) is significant (according to a *t* test), whereas niche conservatism is supported if *d*_b_ > *d*_n_. For each distribution region, the 14 environmental variables, longitude, latitude, and altitude were extracted from the occurrence points and from 1,000 random background points within the background region of each ecotype using the dismo and raster packages in R. The 14 variables were reduced by PCA of the correlation matrix using the ade4 package. Correlations between the reduced PCA axes and the geographic variables (longitude, latitude, and altitude) were examined with a non‐parametric correlation test implemented in perm (Fay & Shaw, 2010). In this study, *d*_n_ and *d*_b_ were calculated as the differences between the mean scores of 75% randomly selected occurrence points of the two niches being compared (*d*_n_) and of the 1,000 background points of the two compared background habitats (*d*_b_), on the reduced PCA axes. The distributions of *d*_b_ and *d*_n_ were generated with 1,000 resamplings, and the mean of *d*_n_ was compared to the 95% confidence interval of *d*_b_ to determine its significance. The significance of the observed divergence between two compared niches (*d*_n_) was determined by a permutation *t* test in perm.

## 3 RESULTS

### 3.1 SNP discovery, genetic diversity, and population structure

Our GBS procedure generated 491.6 million high‐quality reads from 192 individuals. The mean and median numbers of reads per individual were 2.56 million and 2.25 million, respectively. The SAMtools and BCFtools pipeline recovered 393,488 variants; one individual with a high missing rate was discarded. After stringent filtering, we retained 4,077 high‐quality SNPs for the 191 individuals. The mean read depth and mean heterozygous rate per locus were 97× and 22.4%, respectively (Supporting Information Figure S2). The missing rate per SNP was low (mean 7.2%), with 2,960 SNPs (72.6%) genotyped in more than 90% individuals, and 167 out of 191 individuals had missing data for <10% of the SNPs (Supporting Information Figure S2). More importantly, our GBS and bioinformatics strategy removed much repetitive sequences from our data: Of the final 4,077 SNPs, 2,202 (54%) were mapped to genic regions of 886 genes, suggesting the overall high quality of the SNP data.

High genetic diversity was detected in *P. tabuliformis*, with >78% of loci were polymorphic in almost all local populations and the heterozygosity over all samples was 0.225 (Table 1). Using all SNPs (4077) as well as unlinked SNPs (1679), Admixture revealed the split of two southern populations (GY and NS) from the rest under *K *=* *2, and then, two central populations (ZW and HL) were each detected under *K *=* *3 and *K *=* *4, respectively (Supporting Information Figure S3). For *K *=* *5, the 17 populations can be roughly divided into a southern group (two populations: GY and NS), a large northern group (nine populations: DS, FS, JL, LK, LS, NC, SS, TB, and WT), and a western group (four populations: HZ, JZ, QL, and RG), and two small groups each represented by a single population (ZW and HL) (Figure 1). A relatively high degree (14%–49%) of admixture was detected in most of the populations. The two single‐population groups, ZW and HL, were each dominated by population‐specific ancestry. Subdivision under *K *=* *5 seems the most stable and was supported by the Evanno's Δ*K* evaluation (Supporting Information Figure S3c).

Partitioning the genetic variance along the first four PC axes clearly separated all five groups detected by Admixture (Supporting Information Figure S4). All admixed individuals in Admixture occupied intermediate positions within the PCA space. After removing the ZW and HL populations, the remaining 169 individuals from 15 populations became well separated into three groups according to the first two PC axes (Figure 1b). To test whether the population structure revealed by the PCA is explained by a few loci with high genetic differentiation, we excluded 236 SNPs with *F*_ST_ > 0.1 among groups or 46 identified outlier SNPs (*F*_ST_ = 0.117–0.597, see section *Outlier detection*) and recovered the same grouping patterns (data not shown).

Genetic differentiation (*F*_ST_) among the five groups was 0.029, and the differentiation between the south group and the north and west groups (*F*_ST_ = 0.030–0.032; Supporting Information Table S4) was noticeably higher than that between the north group and the west group (*F*_ST_ = 0.006). The two single populations, ZW and HL, were differentiated from the other groups at levels of *F*_ST_ = 0.024–0.046 (Supporting Information Table S4).

A total of 216 individuals were sequenced for three mtDNA segments (Table 1). The combined haplotypes over the three segments identified 10 mitotypes, all of which have been reported in this species before by Wang et al. (2011) (GenBank accession numbers HM467715–HM467734). Among the 10 mitotypes, two were singletons and were removed from RDA. The spatial distribution of the mitotypes is summarized in Figure 1c. Populations from the west and southwest displayed unique haplotype compositions, while populations from the large north shared many haplotypes together. This spatial distribution of diversity corroborated largely with the pattern observed in the GBS data, which divided most of the populations into three geographic groups. On the mtDNA, regional population structure was strong (*F*_ST_ = 0.808) as expected for seed‐dispersed genome.

### 3.2 Demographic history of *P. tabuliformis*

**Figure 2:**
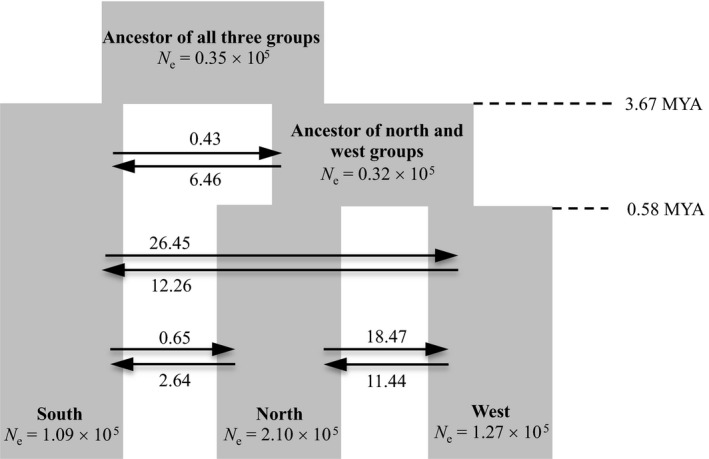
Demographic model of divergence between three major groups of *P. tabuliformis*. Each block represents a current or ancestral population with their estimated effective population size (*N*_e_). Arrows denote the direction of gene flow with the estimated migration rate labeled above or below the arrow. The timings of the two splitting events are indicated in million years ago (MYA)

Among the nine isolation‐with‐migration (IM) models, the one that assumed that the south group diverged first and each group had constant population size after splitting was assigned the highest likelihoods (Akaike's weight, *w*_i_ = 1; Figure 2 and Supporting Information Figure S1). Based on this scenario, the selection for the best‐fitting model well projected the observed SFS (Supporting Information Figure S5), suggesting that the recovered demographic parameters are good estimates of the population history. Results were similar if we excluded singleton from SFS (data not shown). The estimated divergence time between the south group and the ancestral population of the north and west groups was 3.67 MYA (95% CI: 8.04–2.65 MYA; Supporting Information Table S5). The divergence time between the north and west groups was estimated at 0.58 MYA (95% CI: 0.71–0.55 MYA; Supporting Information Table S5). The current effective population sizes of the south, north, and west groups were estimated to be 1.09 × 10^5^, 2.10 × 10^5^, and 1.27 × 10^5^, respectively. Gene flow among the groups was high particularly between south and west (12–26 migrants per generation), and north and west (11–18 migrants per generation). It should be noted that with an effective population size in the order of 10^5^ and a long generation time of 50 years, the divergence among the three groups is still rather recent on a coalescent timescale, 0.12–0.73 *N*_e_ generations. The recent divergence (on coalescent timescale) and high rate of gene flow among populations could explain the observed low genetic differentiation among them (*F*_ST_ = 0.006–0.032; Supporting Information Table S4).

### 3.3 Outlier detection

Using the 4,077 input SNPs, Pcadapt detected 115 (2.82%) outlier SNPs, and Bayenv2 identified 228 (5.59%) SNPs significantly associated with at least one of the 14 examined environmental variables. Because outliers detected by only one method may contain a high proportion of false positives, we chose the 46 SNPs detected by both approaches as more robust adaptive outliers (Supporting Information Table S6). The *F*_*ST*_ over these 46 SNPs ranged between 0.117 and 0.597 among the five groups of populations. These SNPs were significantly associated with temperature (33 SNPs), water availability (13 SNPs), soil type (10 SNPs), and UVB (3 SNPs) variables (Supporting Information Table S6). The potential functions of the genes carrying these SNPs were deduced from annotations of their homologs in the *P. taeda* genome. We found 27 of the 46 SNPs were located within functional genes, including ABC transporter B family, MYB family transcription factors, and armadillo (ARM)‐repeat superfamily (Supporting Information Table S6).

### 3.4 Partitioning genomic variation

We used RDA to partition the population genetic variation into three causal components: environment, geography, and colonization history from multiple refugia. First, we conducted RDA using 3,780 neutral SNPs (i.e., all 4,077 SNPs excluding outliers detected by either Pcadapt or Bayenv2). In the uncorrected RDA, environment and geography explained small but significant proportions of genetic differentiation in *P. tabuliformis*, as measured by adjusted *R* ^2^ (*R* ^2^ = 0.019–0.031, *p *<* *0.05; “combined fractions” in Table 2), but the mtDNA component was not significant (*R* ^2^ = 0.033, *p *>* *0.05). To further disentangle the contributions of climate and geography to among‐population variation, we performed a series of partial RDA (“individual fractions” in Table 2). We found 3.5% of the among‐population variation could be explained by the two components and their joint effect (“total explained” in Table 2), leaving a large proportion (96.5%) of the variation unexplained. Both environment (constrained by geography) and geography (constrained by environment) alone explained little of the observed genomic variation (1.6% and 0.5%, respectively; *p *>* *0.05).

**Table 2:** Redundancy analyses (RDAs) to partition among‐population genetic variation (F) in *Pinus tabuliformis* into environment (env.), geography (geog.), mitochondrial DNA (mito.), and their combined effects, shown in the table as measured by adjusted *R* ^2^

	Neutral SNPs (3780 SNPs)	Bayenv2 & Pcadapt (46 SNPs)	Pcadapt (115 SNPs)	Bayenv2 (228 SNPs)
Combined fractions				
F~env.	0.031*	0.354**	0.314**	0.211*
F~geog.	0.019*	0.150**	0.162**	0.093*
F~mito.	0.033^ns^	0.104^ns^	0.089^ns^	0.055^ns^
Individual fractions				
F~env.|geog.	0.016^ns^	0.207*	0.168**	0.126*
F~geog.|env.	0.005^ns^	0.004^ns^	0.015^ns^	0.008^ns^
F~env. + geog.	0.014	0.147	0.146	0.085
Total explained	0.035	0.358	0.329	0.219
Total unexplained	0.965	0.642	0.671	0.781

Notes

F = dependent matrix of population allele frequencies; RDA tests are of the form: F~independent matrices | covariate matrices. env. = environment (PC1 and PC2); geog. = geography (y); mito. = mitochondrial DNA (PCs 1‐4). The number of SNPs for each dataset is given in parentheses.

Total explained = total adjusted *R* ^2^ of individual fractions.

*p <* *0.05; **p <* *0.01; ^ns^not significant. Significance of confounded fractions between environment and geography was not tested.

We then performed RDA using outlier SNPs and found again insignificant impact of mtDNA but much greater contribution of environment and geography, which jointly explained 21.9%–35.8% of the variation. The contribution of environment and geography was even higher on the 46 outlier SNPs identified jointly by both Pcadapt and Bayenve2. In the uncorrected RDA, both environment and geography were significant and explained 35.4% and 15.0% of the variation, respectively. In the corrected RDA, environment exclusively explained 20.7% (*p *<* *0.05) of the differentiation in the 46 SNPs, but geography alone was insignificant (0.4%, *p *>* *0.05).

### 3.5 Niche divergence and species distribution modeling

**Figure 3:**
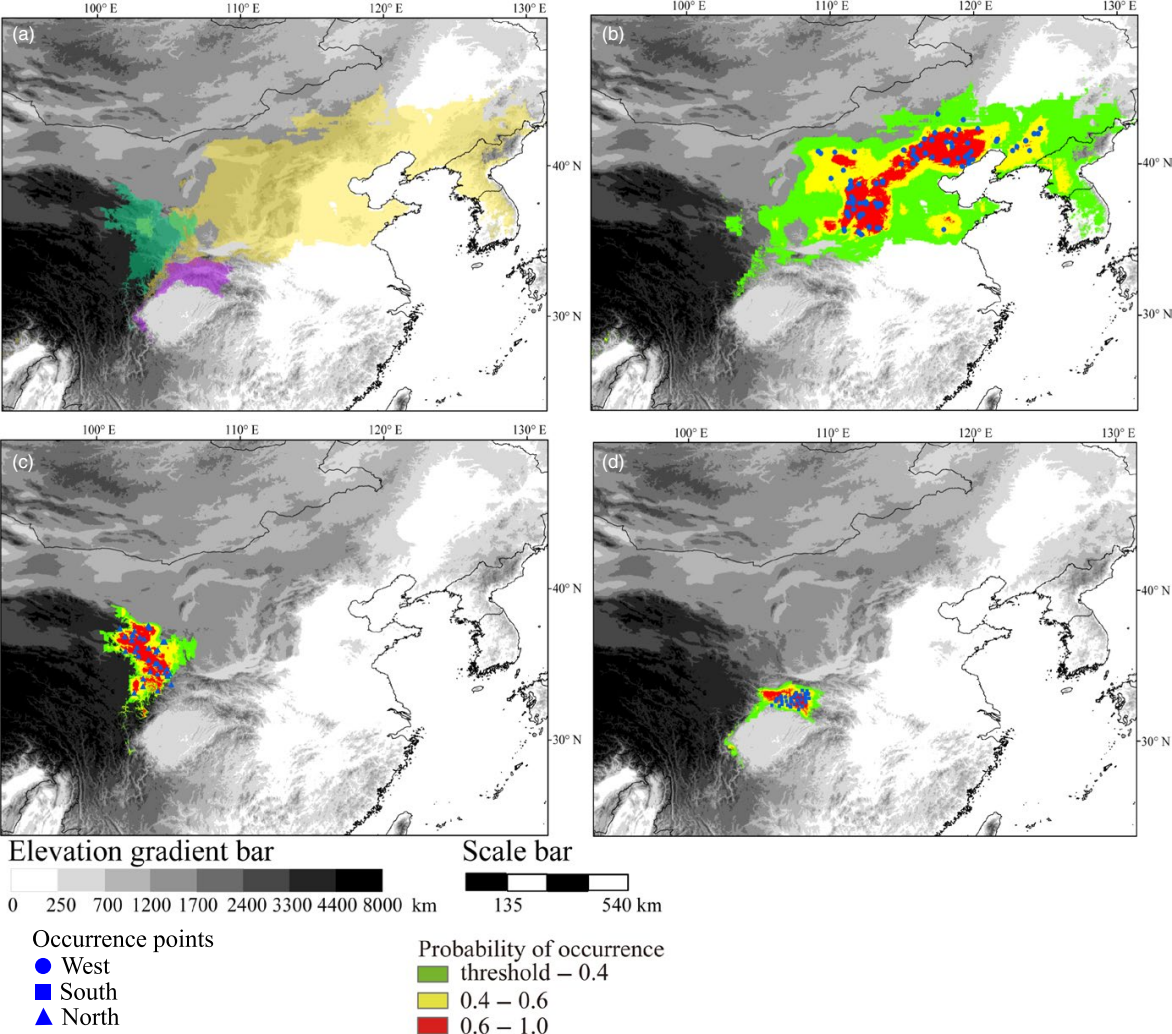
Predicted distributions for three *P. tabuliformis* groups: (a) The three distribution maps, delimited with the threshold obtained by minimizing the sum of sensitivity and specificity on the test data, are superimposed to illustrate the degree of overlap. The distributions of west, north, and south are shown in green, gold, and purple, respectively. (b) North group, (c) west group, and (d) south group. Species occurrence points used in the modeling are shown. The thresholds used for species distribution models of north, south, and west are 0.234, 0.156, and 0.221, respectively

The ecological niche modeling was conducted to define the patterns of environmental variation within and between the three groups of *P. tabuliformis* to evaluate how niche differentiation promotes local adaptation in this species. Based on known occurrence records, we generated a distribution map predicting the areas in which each group of populations might occur (Figure 3). The niche modeling accurately predicted the distribution of the three groups (north, south, and west), with all training and test AUC values greater than 0.99 (*p *<* *0.0001). The predicted distributions of the three groups were generally consistent with the actual geographic ranges of their occurrences. The niche similarity between the north and west groups (Schoener's *D *=* *0.11 and Warren's *I *=* *0.30) was the highest of the three niche pair comparisons (Supporting Information Table S7). Using background tests to examine whether the observed niche divergence between population occurrence locations is larger or smaller than the environmental differences in the surrounding space, we found that all pairs of groups support significant niche differentiation (Supporting Information Table S7).

Within the framework of background testing, multivariate analysis further tests for divergence along independent niche axes. Four to five axes were identified (each with an eigenvalue >1) that explained more than 80% of the total variation in each of the three pairwise comparisons (Table 3). In all three pairwise comparisons, the reduced niche axes were primarily associated with heat, UV, water availability, frost frequency, and soil property. In all pairwise tests, divergence was supported across all niche axes (i.e., *d*_n_ > *d*_b_ and *d*_n_ is significant), strongly suggesting the involvement of ecological factors in population divergence. The north group occupies a niche with an arid climate of strong seasonality. The south group is less seasonal, more mild, and moist than north, while the west group is characterized as having strong UVB and low heat energy (Table 3 and Supporting Information Table S2). Taken together, our results suggest that each of the climatic clusters identified here represents a niche with unique ecological characteristics.

**Table 3:** Divergence on independent niche axes between niche pairs in the *Pinus tabuliformis* distribution

	North vs. South				North vs. West					South vs. West			
	PC1	PC2	PC3	PC4	PC1	PC2	PC3	PC4	PC5	PC1	PC2	PC3	PC4
*d*_n_ a	0.30	0.44	0.42	0.72	0.31	0.44	0.41	0.7	0.94	0.29	0.43	0.41	0.7
*d*_b_ b (95% null distribution)	0–0.25	0.01–0.27	0–0.16	0–0.13	0–0.24	0–0.26	0–0.14	0–0.14	0–0.11	0–0.24	0–0.23	0–0.14	0–0.13
Top‐loading variable^b^	bio1, bio3, bio4, GDD, UVB1	bio2, bio14	FRS, WET	bio5	bio1, bio3, GDD, UVB1	bio2, bio14	FRS	bio5	SC	bio1, bio4, GDD, UVB1	bio2, biao14	FRS, WET	bio5, SpH
% variance explained	35.36	21.13	13.11	11.81	35.32	21.14	13.11	11.83	6.85	35.57	20.74	13.19	11.75
Biological interpretation	Temperature, UV	Temperature, water	Frost, water	Temperature	Temperature, UV	Temperature, water	Frost	Temperature	Soil	Temperature, UV	Temperature, water	Frost, water	Temperature, soil
Correlation longitude	−0.24^c^	0.36^c^	0.05^c^	−0.21^c^	−0.24^c^	0.36^c^	0.05^c^	−0.21^c^	−0.30^c^	−0.24^c^	0.36^c^	0.05^c^	−0.21^c^
Correlation latitude	−0.92^c^	0.06^c^	−0.19^c^	0.12^c^	−0.92^c^	0.06^c^	−0.19^c^	0.12^c^	−0.18^c^	−0.92^c^	0.06^c^	−0.19^c^	0.12^c^
Correlation altitude	−0.15^c^	−0.35^c^	0.22^c^	−0.67^c^	−0.16^c^	−0.35^c^	0.22^c^	−0.67^c^	0.53^c^	−0.16^c^	−0.35^c^	0.22^c^	−0.67^c^

a Significant niche differentiation was detected (i.e., *d*_n_ > *d*_b_ and *d*_n_ is significant, *t* test *p *<* *0.001) for all principal component (PC) axes shown.

b See Supporting Information Table S2 for definitions of the variables.

c *p *<* *0.001 for correlation between PC axes and geographic variables.

## 4 DISCUSSION

### 4.1 Phylogeographic history of *P. tabuliformis*

Our demographic simulations suggested that the divergence of the south, north, and west groups of *P. tabuliformis* initiated in the late Neogene era. The first splitting event between the south group and the ancestral population of the north and west groups occurred 3.67 MYA (95% CI: 8.04–2.65 MYA). This event coincided with the uplift of the Qinling Mountains from the late Miocene and early Pliocene (7.28–3.60 MYA) to the Pleistocene (Ge et al., 2012). The Qinling Mountains are an east–west orientated mountain chain in central China, extending over 1,500 km and rising to 2,000–3,000 m above sea level (Rost, 1994). This mountain range has become a significant physical obstacle for the northward movement of the seasonal monsoon since the late Miocene, and it created an important climate boundary that resulted in a warm‐humid subtropical climate to the south of the mountain range and a cool‐dry temperate climate to the north (Rost, 1994). In addition, the Qinling Mountains also function as a strong barrier to the dispersal of pollen and seeds, thus defining the phylogeography of many plant species in central China (e.g., Guo et al., 2014; Tian et al., 2009; Yuan et al., 2012).

The divergence between the north and west groups occurred 0.58 MYA (95% CI: 0.71–0.55 MYA), coinciding with the Pleistocene glaciations in northern and western China including regions surrounding the eastern Tibetan Plateau (Li et al., 2001; Zhou, Wang, Wang, & Xu, 2006). Despite the debate on exact timing of the uplift across the Tibetan Plateau (Renner, 2016), it is accepted that Pleistocene glaciations have affected the distribution of plant taxa in these regions (Qiu, Fu, & Comes, 2011; Renner, 2016). The environmental changes during Pleistocene could have disrupted the continuous distribution of *P. tabuliformis* and promoted genetic differentiation between north and west groups. The west group is distributed along the northeastern edge of the Tibetan Plateau (Figure 1). Molecular evidence from other conifers suggests that a glacial refugium was located at lower elevations in this region (Meng et al., 2007; Wang et al., 2011), and populations of *P. tabuliformis* in this area represent a colonization lineage from the local refugium after the last glaciations as revealed by mtDNA patterns in our study and previous studies (Chen et al., 2008; Hao et al., 2018).

Geological studies suggest that Pleistocene glaciations in northern and western China were geographically limited to high mountains and that neither region was extensively glaciated (Zhou et al., 2006). Ecological niche modeling of the LGM distribution of *P. tabuliformis* largely overlaps with its current range (Hao et al., 2018). This would imply that *P. tabuliformis* has been relatively stable since the Pleistocene, which corroborates our coalescent simulation results that revealed large effective population sizes in all three genetic groups and no significant recent population expansion. In addition to the three major geographic groups, our current GBS data and previous cytoplasmic DNA data and pollen records suggest the presence of multiple micro‐refugia for *P. tabuliformis* (Hao et al., 2018). The distinct and localized lineages, for example, HL and ZW, are likely relic populations preserved from micro‐refugia.

The high diversity and large effective population sizes of *P. tabuliformis* could be also contributed by introgression from relatives currently or previously parapatric with *P. tabuliformis*. Interspecific hybridization has been well documented in Asian *Pinus* species (e.g., Wang et al., 2011; Yang et al., 2015; Zhou et al., 2017) and shared mito‐ and chlorotypes are commonly found in zones of contact or via long‐distance dispersal (Wang et al., 2011; Yang et al., 2015). A genomewide scan together with sampling of all neighboring sister species would be required to assess the impact of introgression on genome diversity in *P. tabuliformis*.

There are a few caveats in our estimation of demographic parameters. First, the demographic models are likely too simplified to track the real divergence history of the species. Second, a few localized lineages of *P. tabuliformis* identified by mtDNA (Hao et al., 2018) are not included in our simulations. These lineages, including HL and ZW, represent populations recovered from micro‐refugia and are important features of the evolutionary history of the species. Thus, a full demographic history of *P. tabuliformis* can only be established through denser sampling of populations across its full distribution, and through more powerful statistical models applied on genome‐wide data. Third, the time frame converted from coalescent‐scaled estimates depends on the mutation rate and generation time; both are difficult to estimate accurately for conifer trees. In this study, we assumed a mutation rate per year of 7.0 × 10^−10^ for the genus *Pinus* based on Willyard et al. (2007). Other mutation rates ranging from 4.16 × 10^−10^ (De La Torre, Li, Van de Peer, & Ingvarsson, 2017; Gao et al., 2012) to 13.1 × 10^−10^ (Willyard et al., 2007) per site per year are also proposed for pine species. Defining the generation time of *Pinus* is also difficult, because pine species have long maturation time and continue to produce seeds for decades without a peak of fertility. In its central distribution, *P. tabuliformis* reaches seed production at around age 20 (Li, Wang, & Li, 2012). A generation time of 50 years and a mutation rate per year of 7.0 × 10^−10^ can be considered reasonable parameters for *P. tabuliformis*. When using the initial reproductive age of *P. tabuliformis* as the generation time, which usually underestimates the actual generation time, and a upper bound of the mutation rate (13.1 × 10^−10^ per site per year), the estimated time of the two splitting events in *P. tabuliformis* is 0.78 MYA (between south group and the ancestor of north and west groups) and 0.12 MYA (between north and west groups), which were still much earlier than the LGM (0.02 MYA; Hewitt, 2000). Hence, we conclude that the divergence in *P. tabuliformis* predated the LGM and has been affected by major geological events and subsequent climatic changes during Pliocene–Pleistocene.

### 4.2 Environmental adaptation in *P. tabuliformis*

Partitioning of the divergence over 3,780 neutral SNPs into three causal components (IBC, IBE, and IBD) revealed that only 3.5% of the among‐population variation could be explained by climate and geography, leaving 96.5% of the genetic variation unexplained. In contrast, a significant 20.7% of the variation in a group of 46 outliers was attributed to climate and only 0.4% to geography. These results improve our confidence in the outlier SNPs we identified in this study. The absence of IBD in *P. tabuliformis* is not unexpected, firstly due to the extensive gene flow among populations, and secondly because the effects of IBD and IBE are usually not mutually exclusive (Orsini et al., 2013), as shown in the 46 outliers for which 14.7% of the genetic variation was confounded by IBD and IBE.

In contrast to a study that reports a predominant effect of IBC on genomic differentiation in two white pine species *P. strobus* and *P. monticola* in North America (Nadeau et al., 2016), we detected insignificant impact of IBC on both neutral and outlier variation. Dissecting IBC from IBD and IBE is difficult because their effects are often confounded (Orsini et al., 2013), and IBC may override the signals of IBD and IBE in species with a recent and rapid colonization history. The two North American pines recolonized most of their range recently through long‐distance postglacial colonization from two main refugia, which covered a large geographic range (Nadeau et al., 2015), while *P. tabuliformis* were preserved in multiple refugia from which genetic lineages expanded locally. These very different expansion histories might explain the contrasting roles of IBC in these species. The absence of IBC in *P. tabuliformis* could also be due to the difficulty of finding a variable independent of the nuclear genome that can be used to track IBC on population structure in the nuclear genome. In this study, we utilized the maternally inherited mtDNA information as a proxy for colonization, but even these markers could be subject to subsequent migration so that they do not necessarily reflect the colonization history that well. The insignificant IBC in *P. tabuliformis*, as reflected by mtDNA, is likely due to the high rates of gene flow among populations, which have eroded much of the maternal history from the nuclear genome. The incomplete sampling of all potential glacial refugia might also have limited our ability to detected IBC in *P. tabuliformis* if these local populations show clear correspondence between mtDNA and nuclear DNA variations.

The genomic signature of IBE as revealed by RDA in *P. tabuliformis* is similar to that found in *P. strobus* (Nadeau et al., 2016), but the amount of variation explained by environment on outlier loci was much greater in *P. tabuliformis*. This signature, however, is not observed in *P. monticola* (Nadeau et al., 2016). The signature of IBE can be generated by natural selection among populations locally adapted to different environments (Nachman & Payseur, 2012; Wang & Bradburd, 2014). Depending on the evolutionary history and the rate of recombination, a pattern of IBE can be observed either locally as outlier loci or spread out to broader genomic regions due to genome hitchhiking (Nachman & Payseur, 2012; Feder et al 2012). We expect that different phylogeographic histories of pine species would result in different patterns of genetic differentiation across their genomes. Unlike the long divergence history of *P. tabuliformis* in distinct niches, which is expected to facilitate adaptive divergence, the population history of most plants in Europe and North America is characterized by their recent colonization after the LGM (Hewitt, 2000). Thus, there may have not been sufficient time for strong adaptive signatures in the genome to evolve, as the population structure in the two white pine species is primarily shaped by periodic isolation and range expansion.

In the face of gene flow, 46 SNPs stands out as significantly associated with local environments. Annotation of the 46 outliers identified 27 genes involved in plant stress responses, which may have mediated environmental adaptation in *P. tabuliformis*. Alternatively, these genes carrying the outliers represent intrinsic genetic incompatibility loci (endogenous barriers) rather than loci involved in local adaptation (exogenous barrier). Bierne, Welch, Loire, Bonhomme, and David (2011) suggest that endogenous genetic barriers, resulting from processes independent from environment selection, accumulate during population divergence in isolation. Distinguishing their relative roles in promoting population differentiation is difficult, but low genetic incompatibility between sister pine species and between populations within species (Vasilyeva & Goroshkevich, 2013; Zhao et al., 2014) does not support a strong contribution of endogenous barriers in shaping intraspecific population structure in pines. On the other hand, our niche modeling revealed strong environmental differentiation between the three genetic groups (north, west, and south) of *P. tabuliformis,* with each group occupying a well‐defined geoclimatic zone. The northern niche is characterized by a continental and arid climate with strong seasonal variation, while the southern niche is characterized by a more mild and moist climate with less seasonality. The western niche is characterized as having high UV exposure and low temperatures. Taken together, these results lead us to the conclusion that each of the three groups of *P. tabuliformis* occupies a different environmental niche, and such niche diversity could have important consequences for local adaptation in *P. tabuliformis*. However, given the polygenic nature of climate adaptation in trees, the genomic signature of adaptation is expected to be weak and difficult to detect without comprehensive genome‐scan.

## 5 CONCLUSIONS AND IMPLICATIONS

This study provides the first genomewide investigation into the diversity and evolutionary history of *P. tabuliformis*. Our attempt to incorporate GBS, mtDNA, geography, and climate data in RDA offers a novel approach to understanding the complex interactions of IBE, IBD, and IBC and their impacts on population genomic structure. Our results show that most of the observed genomic structure in *P. tabuliformis* is affected by neutral and stochastic processes, and the impact of maternal history has been eroded by gene flow. The signature of IBE is not clearly visible, which is in line with the expectation for quantitative traits in a large complex genome background. Nonetheless, we recovered support for environment‐driven genetic differentiation in a group of 46 outlier loci, which represent 1% of the survey SNPs or 2% of the SNPs from genic regions of 886 genes, in the face of strong gene flow. These findings from limited sampling of the genome represent an important first step in understanding the origin and maintenance of genetic variation in *P. tabuliformis*. Based on these results, further genome scans can be optimized to better characterize adaptive genetic diversity in this species.

Deep sampling of the genome is necessary to uncover the fraction of the genome involved in adaptation due to low linkage disequilibrium in conifers (Tiffin & Ross‐Ibarra, 2014). GBS, if carefully designed, can be a cost‐effective method for generating genome‐wide data for population genetic investigations. In this study, we first optimized the GBS library preparation strategy to reduce the presentation of repetitive regions in sequencing library. Secondly, we applied a reference‐guided mapping and variance calling strategy, in which repetitive regions are further filtered out from our data. This approach makes GBS an attractive and effective genome‐scan method for conifer species, especially when genome resequencing and RNA‐seq on large number of individuals are economically constrained.

The discovery of major genetic groups in *P. tabuliformis* and the association of these groups to distinct niches will inform forest management decisions and seed transfer guidelines following ecological zones. Tree breeding programs are established for different climate conditions, and our ecological niche characterization over the species range provides a good reference for deciding breeding zones. The unique refugium populations identified by this study call for additional investigations into their breeding and conservation values.

## DATA ARCHIVING STATEMENT

Raw Illumina reads are available in the NCBI SRA database under project accession ID: SRP153314.
