# Supplementary material for: Combining mitochondrial and nuclear genome analyses to dissect the effects of colonization, environment, and geography on population structure in Pinus tabuliformis
Source: Evol Appl. 2018 Sep 24;11(10):1931–45. doi: 10.1111/eva.12697 (PMC6231471; doi:10.1111/eva.12697)
Supplement: Supplementary file 1 [file EVA-11-1931-s001.pdf]

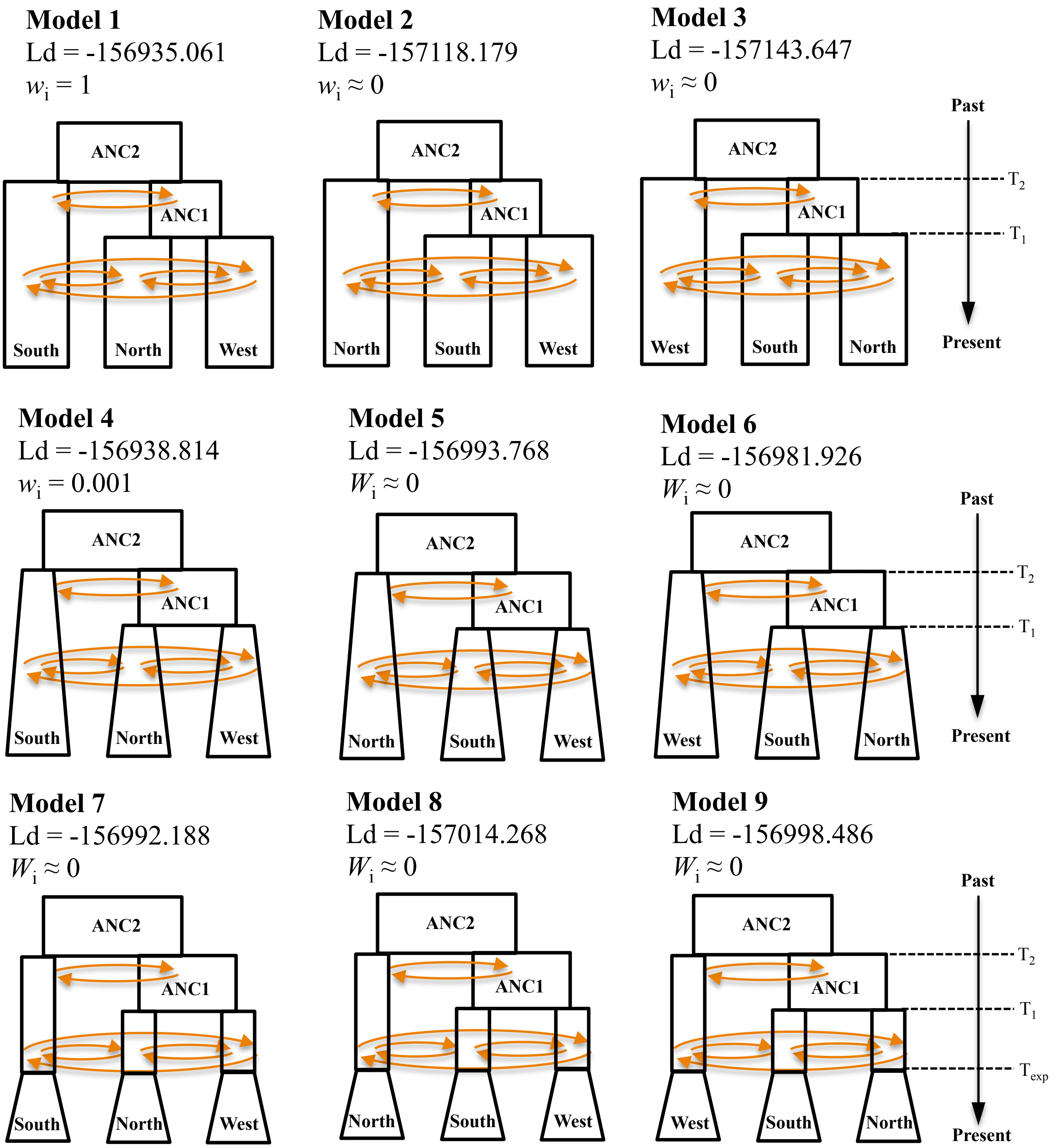

Fig. S1. Nine demographic scenarios with different orders of branching: the south group diverged first (models 1, 4 and 7), the north group diverged first (models 2, 5 and 8) and the west group diverged first (models 3, 6 and 9). In models 1 - 3, all groups have constant population sizes after splitting. Models 4 - 6, all groups experienced exponential population size change after splitting. Models 7 -9, all groups expanded exponentially at a recent time after splitting of all groups. Likelihood of each model was estimated by fastsimcoal2. Ld is the maximum of the likelihood values among the 50 independent runs for each model, and wi is the relative likelihood calculated according to Excoffer et al. (2013).

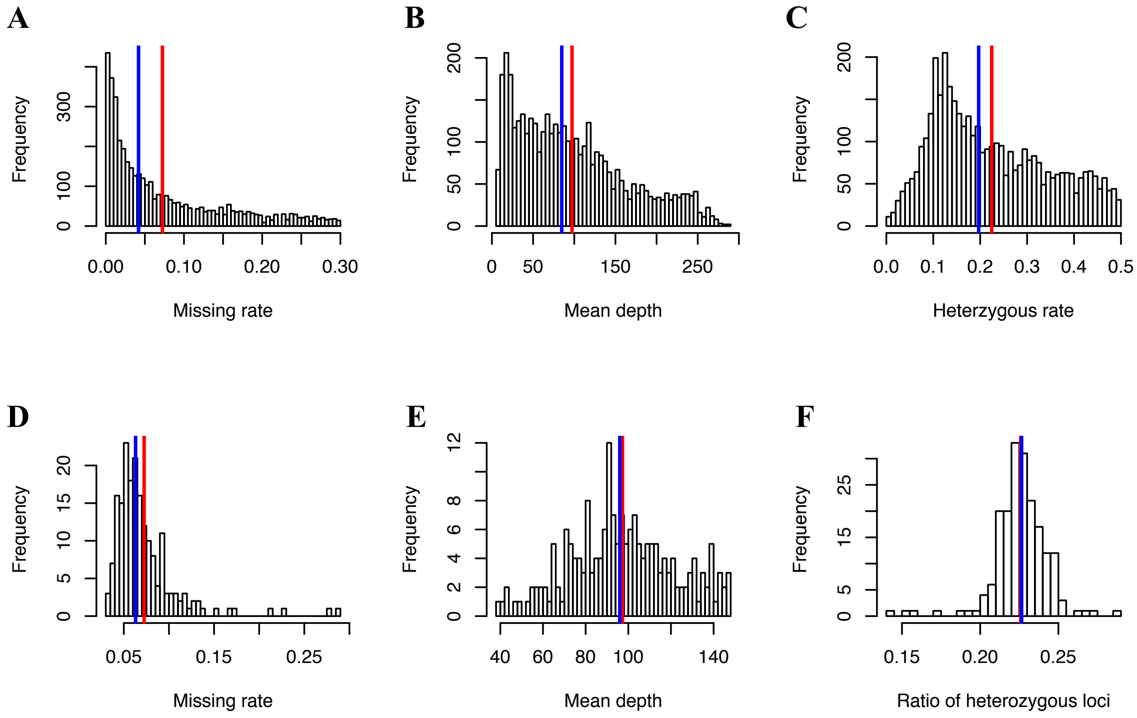

Fig. S2. Histgram of GBS data quality. A – C show distribution of missing rate, mean sequencing depth and heterozygous rate per site, respectively. D – F show distribution of missing rate, mean sequencing depth and heterozygous rate per individual, respectively. Red and blue vertical lines indicate the mean and median value of the distribution, respectively.

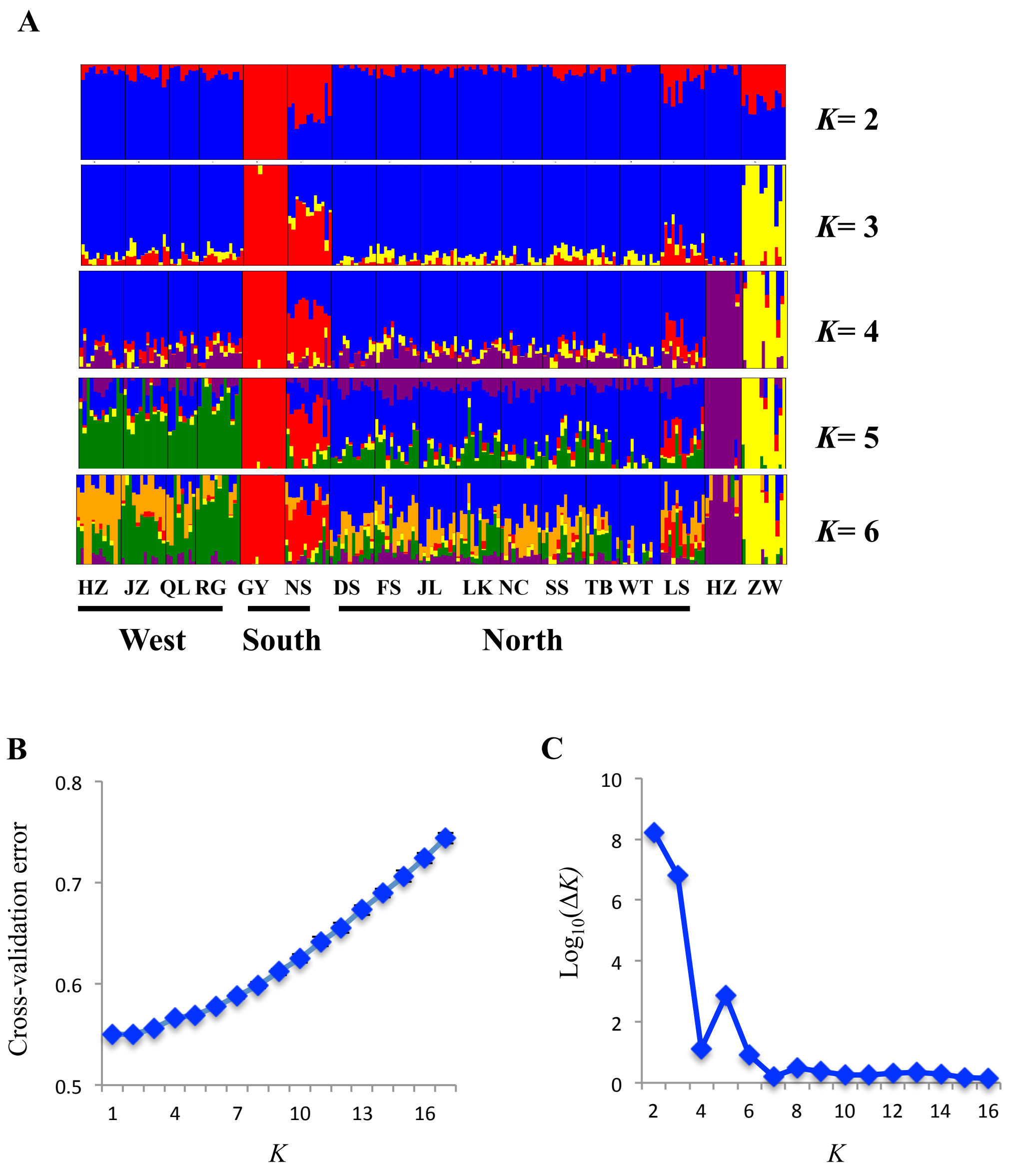

Fig. S3. Admixture analyses on GBS data for *P. tabuliformis*. (A) Admixture assignment for 17 populations with $K = 2 – 6$. (B) Plot of Admixture Cross-validation (CV) error. (C) Plot of ΔK values estimated from log-likelihood.

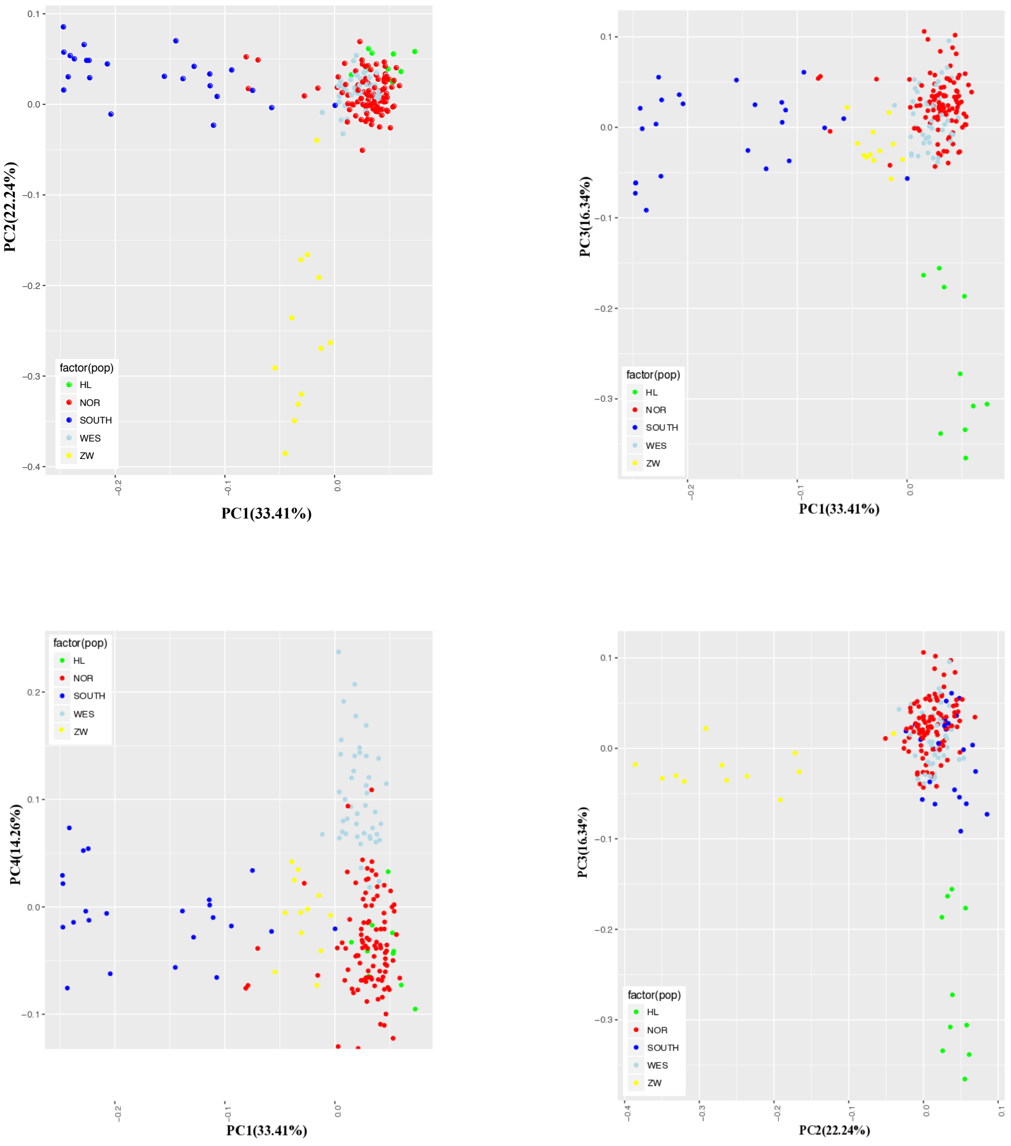

Fig. S4. PCA plots of the GBS variation in 17 populations of *P. tabuliformis*. Patterns of genetic divergence along PC1–PC4 are presented.

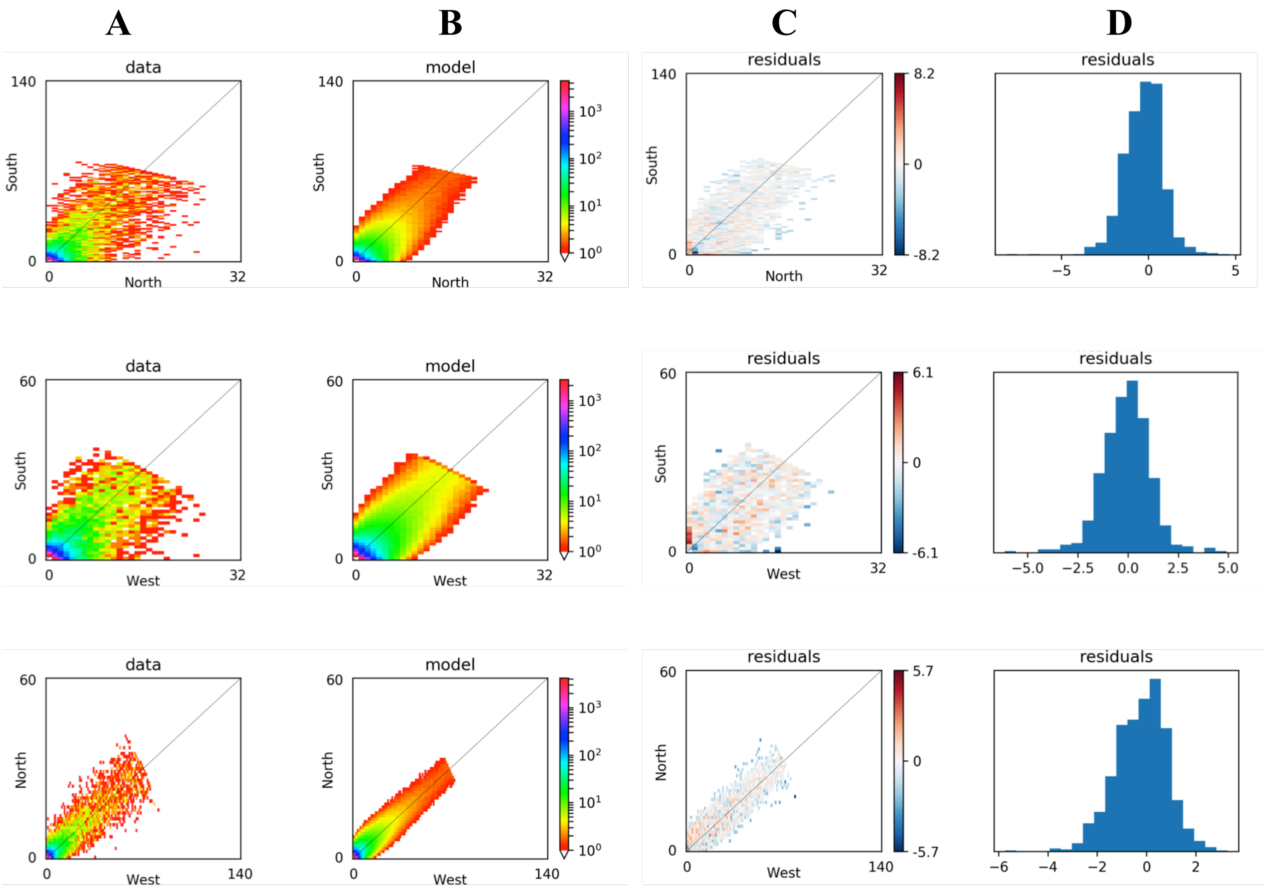

Fig. S5. The goodness of fit of the best-fitting demographic model inferred by fastsimcoal2. For each pair of groups, south *vs*. north (top), south *vs*. west (middle) and north *vs*. west (bottom), the observed joint SFS (data, A) is compared with expected joint SFS (model, B), and the residuals between data and model are plotted in a colormap (C) and a histogram (D). In the colormap, red or blue residuals indicate that the model predicts too many to too few alleles in a given cell, respectively.
