# Supplementary material for: Combining mitochondrial and nuclear genome analyses to dissect the effects of colonization, environment, and geography on population structure in Pinus tabuliformis
Source: Evol Appl. 2018 Sep 24;11(10):1931–45. doi: 10.1111/eva.12697 (PMC6231471; doi:10.1111/eva.12697)
Supplement: Supplementary file 2 [file EVA-11-1931-s002.pdf]

Table S1 Parameters and search ranges for nine isolation-with-migration models.

Parameters	Model 1	Model 2	Model 3	Model 4	Model 5	Model 6	Model 7	Nodel 8	Model 9
N_1	$10^4 - 10^6$	$10^4 - 10^6$	$10^4 - 10^6$	$10^4 - 10^6$	$10^4 - 10^6$	$10^4 - 10^6$	$10^4 - 10^6$	$10^4 - 10^6$	$10^4 - 10^6$
N_2	$10^4 - 10^6$	$10^4 - 10^6$	$10^4 - 10^6$	$10^4 - 10^6$	$10^4 - 10^6$	$10^4 - 10^6$	$10^4 - 10^6$	$10^4 - 10^6$	$10^4 - 10^6$
N_3	$10^4 - 10^6$	$10^4 - 10^6$	$10^4 - 10^6$	$10^4 - 10^6$	$10^4 - 10^6$	$10^4 - 10^6$	$10^4 - 10^6$	$10^4 - 10^6$	$10^4 - 10^6$
N_{1a}	na	na	na	$10^4 - 10^6$	$10^4 - 10^6$	$10^4 - 10^6$	$10^4 - 10^6$	$10^4 - 10^6$	$10^4 - 10^6$
N_{2a}	na	na	na	$10^4 - 10^6$	$10^4 - 10^6$	$10^4 - 10^6$	$10^4 - 10^6$	$10^4 - 10^6$	$10^4 - 10^6$
N_{3a}	na	na	na	$10^4 - 10^6$	$10^4 - 10^6$	$10^4 - 10^6$	$10^4 - 10^6$	$10^4 - 10^6$	$10^4 - 10^6$
N_{anc1}	$10^4 - 10^6$	$10^4 - 10^6$	$10^4 - 10^6$	$10^4 - 10^6$	$10^4 - 10^6$	$10^4 - 10^6$	$10^4 - 10^6$	$10^4 - 10^6$	$10^4 - 10^6$
N_{anc2}	$10^4 - 10^6$	$10^4 - 10^6$	$10^4 - 10^6$	$10^4 - 10^6$	$10^4 - 10^6$	$10^4 - 10^6$	$10^4 - 10^6$	$10^4 - 10^6$	$10^4 - 10^6$
T_1	$10^4 - 10^6$	$10^4 - 10^6$	$10^4 - 10^6$	$10^4 - 10^6$	$10^4 - 10^6$	$10^4 - 10^6$	$T_{exp} + (10^4 - 10^6)$	$T_{exp} + (10^4 - 10^6)$	$T_{exp} + (10^4 - 10^6)$
T_2	$T_1 + (10^4 - 10^7)$	$T_1 + (10^4 - 10^7)$	$T_1 + (10^4 - 10^7)$	$T_1 + (10^4 - 10^7)$	$T_1 + (10^4 - 10^7)$	$T_1 + (10^4 - 10^7)$	$T_1 + (10^4 - 10^7)$	$T_1 + (10^4 - 10^7)$	$T_1 + (10^4 - 10^7)$
T_{exp}	na	na	na	na	na	na	$10^4 - 10^6$	$10^4 - 10^6$	$10^4 - 10^6$
$M_{0->1}$	$10^{-6} - 0.1$	$10^{-6} - 0.1$	$10^{-6} - 0.1$	$10^{-6} - 0.1$	$10^{-6} - 0.1$	$10^{-6} - 0.1$	$10^{-6} - 0.1$	$10^{-6} - 0.1$	$10^{-6} - 0.1$
$M_{1->0}$	$10^{-6} - 0.1$	$10^{-6} - 0.1$	$10^{-6} - 0.1$	$10^{-6} - 0.1$	$10^{-6} - 0.1$	$10^{-6} - 0.1$	$10^{-6} - 0.1$	$10^{-6} - 0.1$	$10^{-6} - 0.1$
$M_{0->2}$	$10^{-6} - 0.1$	$10^{-6} - 0.1$	$10^{-6} - 0.1$	$10^{-6} - 0.1$	$10^{-6} - 0.1$	$10^{-6} - 0.1$	$10^{-6} - 0.1$	$10^{-6} - 0.1$	$10^{-6} - 0.1$
$M_{2->0}$	$10^{-6} - 0.1$	$10^{-6} - 0.1$	$10^{-6} - 0.1$	$10^{-6} - 0.1$	$10^{-6} - 0.1$	$10^{-6} - 0.1$	$10^{-6} - 0.1$	$10^{-6} - 0.1$	$10^{-6} - 0.1$
$M_{1->2}$	$10^{-6} - 0.1$	$10^{-6} - 0.1$	$10^{-6} - 0.1$	$10^{-6} - 0.1$	$10^{-6} - 0.1$	$10^{-6} - 0.1$	$10^{-6} - 0.1$	$10^{-6} - 0.1$	$10^{-6} - 0.1$
$M_{2->1}$	$10^{-6} - 0.1$	$10^{-6} - 0.1$	$10^{-6} - 0.1$	$10^{-6} - 0.1$	$10^{-6} - 0.1$	$10^{-6} - 0.1$	$10^{-6} - 0.1$	$10^{-6} - 0.1$	$10^{-6} - 0.1$
$M_{0->A}$	$10^{-6} - 0.1$	$10^{-6} - 0.1$	$10^{-6} - 0.1$	$10^{-6} - 0.1$	$10^{-6} - 0.1$	$10^{-6} - 0.1$	$10^{-6} - 0.1$	$10^{-6} - 0.1$	$10^{-6} - 0.1$
$M_{A->0}$	$10^{-6} - 0.1$	$10^{-6} - 0.1$	$10^{-6} - 0.1$	$10^{-6} - 0.1$	$10^{-6} - 0.1$	$10^{-6} - 0.1$	$10^{-6} - 0.1$	$10^{-6} - 0.1$	$10^{-6} - 0.1$

N_1, N_2 and N_3 are current effective population size of groups I, II and III, respectively.

N_{1a}, N_{2a} and N_{3a} are effective population size before expansion for groups I, II and III, respectively.

N_{anc1} is effective population size of ancestror of group II and III, and N_{anc2} is effective population size of all three groups.

T_1, spliting time between groups II and III; T_2, split time between group I and ancestor of group II and III. T_{exp}, time of recent expansion in all three groups.

$M_{i->j}$ migration rate from population i to population j, for example, $M_{0->1}$ represents migration rata from group I to group II.

N_1,N_2, N_3, N_{1a}, N_{2a}, N_{3a}, N_{anc1} and N_{anc2} are in number of haploid individuals, T_1, T_2 and $T_{exp}$are measured in years.

In models 1, 4 and 7, groups I, II and III are south, north and west groups, respectively; in models 2, 5 and 8, groups I, II and III are north, south and west groups, respectively; in models 3, 6 and 9, groups I, II and III are west, south and north groups, respectively;

All parameters are given by a uniform with minimum and maximum.

na, parameter is not included in modle.

Table S2 Environmental parameters used in this study, and the mean (± standard deviation) values in the south, north and west groups of *Pinus tabuliformis*.

Code	Name	Source	South	North	West
Bio1	Annual mean air temperature (°C × 10)	http://www.worldclim.org/	129.727 ± 18.324	71.177 ± 21.938	57.686 ± 26.584
Bio2	Mean diurnal air temperature range (°C × 10)	http://www.worldclim.org/	85.121 ± 4.533	122.062 ± 8.005	124.829 ± 14.664
Bio3	Isothermality (× 100)	http://www.worldclim.org/	27.333 ± 0.924	28.031 ± 1.803	33.457 ± 3.212
Bio4	Air temperature seasonality (× 100)	http://www.worldclim.org/	7783.242 ± 402.64	10965.677 ± 1020.596	8292.429 ± 987.967
Bio5	Max air temperature of the warmest month (°C × 10)	http://www.worldclim.org/	283.121 ± 23.04	270.208 ± 20.421	227.629 ± 34.639
Bio12	Annual precipitation (mm)	http://www.worldclim.org/	956.697 ± 108.723	542.656 ± 128.097	478.943 ± 138.495
Bio14	Precipitation of the driest month (mm)	http://www.worldclim.org/	6.364 ± 1.295	3.906 ± 2.068	2.2 ± 0.797
Bio15	Precipitation seasonality (coefficient of variation)	http://www.worldclim.org/	76.394 ± 4.394	104.552 ± 12.301	85.914 ± 6.094
FRS	Ground-frost frequency (days)	http://www.ipcc-data.org/obs/cru_ts2_1.html	21.101 ± 2.061	29.456 ± 0.748	28.988 ± 1.217
GDD	Growing degree days	http://www.sage.wisc.edu/atlas/index.php	2563.592 ± 217.067	2175.677 ± 340.16	1287.254 ± 398.792
SC	Soil organic carbon (g/cm^3)	http://www.sage.wisc.edu/atlas/index.php	4.514 ± 0.335	4.98 ± 0.678	5.14 ± 1.884
SpH	Soil pH	http://www.sage.wisc.edu/atlas/index.php	6.193 ± 0.142	6.991 ± 0.259	7.173 ± 0.579
WET	Wet-day frequency (days)	http://www.ipcc-data.org/obs/cru_ts2_1.html	12.512 ± 0.439	7.333 ± 0.946	10.162 ± 1.752
UVB1	Annual mean UV-B (J/m^2/day)	http://www.ufz.de/gluv/	2836.242 ± 54.27	2593.615 ± 214.595	3833.886 ± 637.507

Table S3 List of 164 occurrence sites of *Pinus tabuliformis* used for niche modeling.

Sites	Longitude (°E)	Latitude (°N)	Group
Pt1	111.617	37.000	north
Pt2	114.967	40.000	north
Pt3	124.200	42.000	north
Pt4	124.500	42.000	north
Pt5	123.000	41.133	north
Pt6	109.167	40.833	north
Pt7	109.333	40.700	north
Pt8	110.050	39.050	north
Pt9	110.315	40.787	north
Pt10	110.917	39.583	north
Pt11	110.950	35.700	north
Pt12	110.983	35.567	north
Pt13	111.167	36.800	north
Pt14	111.183	36.717	north
Pt15	111.267	36.550	north
Pt16	111.267	37.450	north
Pt17	111.283	38.367	north
Pt18	111.417	38.900	north
Pt19	111.400	38.600	north
Pt20	111.483	37.817	north
Pt21	111.500	38.500	north
Pt22	111.583	37.383	north
Pt23	111.583	37.483	north
Pt24	111.600	37.667	north
Pt25	111.683	40.817	north
Pt26	111.950	35.500	north
Pt27	111.950	35.550	north
Pt28	111.983	38.667	north
Pt29	112.017	35.450	north
Pt30	112.033	36.500	north
Pt31	112.033	36.583	north
Pt32	112.033	37.417	north
Pt33	112.036	36.613	north
Pt34	112.133	36.667	north
Pt35	112.167	35.333	north
Pt36	112.283	36.667	north
Pt37	112.333	37.433	north
Pt38	112.533	37.367	north
Pt39	112.500	37.500	north
Pt40	112.717	35.850	north

Table S3 continued

Sites	Longitude (°E)	Latitude (°N)	Group
Pt41	113.167	37.433	north
Pt42	113.217	38.717	north
Pt43	113.283	35.750	north
Pt44	113.317	37.167	north
Pt45	113.417	35.750	north
Pt46	113.433	35.750	north
Pt47	113.500	37.383	north
Pt48	113.550	37.300	north
Pt49	113.663	38.885	north
Pt50	113.667	38.767	north
Pt51	114.983	39.967	north
Pt52	115.117	40.917	north
Pt53	115.783	40.022	north
Pt54	115.783	40.583	north
Pt55	115.817	40.917	north
Pt56	115.950	40.450	north
Pt57	116.074	40.277	north
Pt58	116.300	41.233	north
Pt59	116.583	41.667	north
Pt60	116.633	40.717	north
Pt61	117.083	41.583	north
Pt62	117.117	40.450	north
Pt63	117.300	42.067	north
Pt64	117.500	43.300	north
Pt65	117.917	35.667	north
Pt66	117.950	40.200	north
Pt67	118.117	41.450	north
Pt68	118.167	40.117	north
Pt69	118.183	42.233	north
Pt70	118.217	40.250	north
Pt71	118.450	41.383	north
Pt72	118.500	41.150	north
Pt73	118.750	41.917	north
Pt74	118.917	40.383	north
Pt75	118.970	42.280	north
Pt76	119.200	39.850	north
Pt77	119.283	42.917	north
Pt78	119.417	40.467	north
Pt79	119.583	41.383	north
Pt80	119.600	40.600	north

Table S3 continued

Sites	Longitude (^oE)	Latitude (^oN)	Group
Pt81	119.650	40.033	north
Pt82	119.700	40.217	north
Pt83	119.817	40.800	north
Pt84	119.833	40.833	north
Pt85	119.950	40.300	north
Pt86	120.300	42.333	north
Pt87	121.583	41.500	north
Pt88	121.650	41.633	north
Pt89	122.717	40.883	north
Pt90	123.633	41.533	north
Pt91	123.900	40.883	north
Pt92	124.667	42.333	north
Pt93	111.000	38.000	north
Pt94	112.083	36.617	north
Pt95	105.833	32.367	south
Pt96	106.100	32.630	south
Pt97	106.210	32.820	south
Pt98	106.350	32.310	south
Pt99	106.480	32.980	south
Pt100	106.500	32.750	south
Pt101	106.550	32.600	south
Pt102	106.790	32.920	south
Pt103	106.840	32.640	south
Pt104	106.950	32.380	south
Pt105	107.140	32.270	south
Pt106	107.150	32.620	south
Pt107	107.160	32.850	south
Pt108	107.230	32.600	south
Pt109	107.390	32.930	south
Pt110	107.420	32.440	south
Pt111	107.530	33.030	south
Pt112	107.538	33.198	south
Pt113	107.610	32.700	south
Pt114	107.710	32.100	south
Pt115	107.760	32.440	south
Pt116	107.840	33.050	south
Pt117	107.900	32.750	south
Pt118	107.965	33.350	south
Pt119	107.980	32.300	south
Pt120	108.170	33.150	south

Table S3 continued

Sites	Longitude (^oE)	Latitude (^oN)	Group
Pt121	108.180	32.920	south
Pt122	108.190	32.810	south
Pt123	108.220	32.550	south
Pt124	108.310	33.320	south
Pt125	108.390	33.120	south
Pt126	108.440	32.840	south
Pt127	102.000	35.883	west
Pt128	101.628	37.392	west
Pt129	101.951	36.846	west
Pt130	102.450	36.610	west
Pt131	102.465	36.956	west
Pt132	102.500	36.959	west
Pt133	102.621	33.120	west
Pt134	102.639	37.241	west
Pt135	102.667	35.867	west
Pt136	102.810	36.140	west
Pt137	102.920	35.580	west
Pt138	102.990	36.800	west
Pt139	103.227	33.559	west
Pt140	103.246	34.060	west
Pt141	103.300	36.700	west
Pt142	103.589	37.584	west
Pt143	103.607	35.301	west
Pt144	103.624	36.091	west
Pt145	103.685	37.475	west
Pt146	103.691	37.448	west
Pt147	103.720	34.560	west
Pt148	104.075	35.064	west
Pt149	104.116	35.845	west
Pt150	104.216	35.117	west
Pt151	104.230	34.380	west
Pt152	104.246	33.268	west
Pt153	104.249	35.984	west
Pt154	104.454	34.841	west
Pt155	104.621	36.513	west
Pt156	104.720	34.440	west
Pt157	104.750	36.500	west
Pt158	104.900	34.729	west
Pt159	104.950	34.230	west
Pt160	105.095	34.380	west

Table S3 continued

Sites	Longitude (^oE)	Latitude (^oN)	Group
Pt161	105.200	33.680	west
Pt162	103.363	33.688	west
Pt163	104.083	33.167	west
Pt164	104.217	33.133	west

Table S4 Weir and Cockerham's mean F_{ST} between (below diagonal) and within (on diagonal) five *Pinus tabuliformis* groups.

	South	North	West	HL	ZW
South	0.016				
North	0.032	0.008			
West	0.030	0.006	0.013		
HL	0.046	0.024	0.025	NA	
ZW	0.045	0.036	0.038	0.059	NA

NA, not appropriable in single population group.

Table S5 Estimated demographic parameters for the south, north and west groups of *Pinus tabuliformis* based on GBS data.

	Point Estimate	2.50%	97.50%
N_1	109046	58199	132260
N_2	210349	185485	258392
N_3	127233	25722	78573
N_{anc1}	32466	14256	59877
N_{anc2}	34555	11546	75870
T_1	0.576	0.551	0.712
T_2	3.666	2.648	8.035
$M_{0\text{->}1}$	0.646	0.299	34.602
$M_{0\text{->}2}$	26.453	1.310	53.521
$M_{1\text{->}0}$	2.642	1.245	33.002
$M_{1\text{->}2}$	18.468	1.255	23.047
$M_{2\text{->}0}$	12.258	0.535	44.218
$M_{2\text{->}1}$	11.440	14.155	26.264
$M_{0\text{->}A}$	0.434	0.078	9.691
$M_{A\text{->}0}$	6.463	0.321	30.186

Point Estimate, 2.5% and 97.5%, are estimated parameters and 95% confidence interval, respectively.

N_1, N_2, N_3, effective population size of south, north and west groups, respectively.

N_{anc1}, effective population size of ancestror of north and west groups; N_{anc2} is effective population size of all three groups.

T_1, split time between north and west groups; T_2, split time between south group and ancestor of north and west groups.

$M_{i\text{->}j}$ population migration rate from group i to group j, for example, $M_{0\text{->}1}$ represents migration rate from south group to north group.

N_1 and N_2 are measured in units of individuals; T_1 and T_2 are measured in million years.

Table S6 List of 46 genes carrying outlier SNPs identified by both Pcadapt and Bayenv2, and descriptive information.

		Temperature (33)							Water availability (13)				Soil (10)		UVB(3)
SNPs	Function	Bio1 (18)	Bio2 (17)	Bio3 (3)	Bio4 (3)	Bio5 (6)	GDD (11)	FRS (16)	Bio12 (9)	Bio14 (5)	Bi	Wet (6)	SC (5)	SpH (5)	UVB1 (3)
C29619294-174	NA	+	+	-	-	-	+	+	-	-	-	-	+	-	-
C29619294-251	NA	+	-	-	-	-	-	-	-	-	-	-	-	-	-
C32026140-3084	NA	+	+	-	-	-	-	+	-	-	-	-	-	-	-
C32218754-12065	NA	+	+	-	+	-	-	+	+	+	+	+	-	+	-
scaffold257800-11915	NA	-	-	-	-	-	-	-	+	+	-	-	-	-	-
scaffold298926-151817	3-ketoacyl-CoA thiolase 2, peroxisomal, Thiolase N-terminal,Thiolase C-terminal	-	-	-	-	+	-	-	-	-	-	-	-	-	-
scaffold30036.2-43632	Protein phosphatase 2C (PP2C)	-	-	-	-	-	-	-	-	-	-	+	-	-	-
scaffold377079.1-91528	myb family transcription factor	-	+	-	-	-	-	-	-	-	-	-	-	-	-
scaffold463926-98346	uninformative,no protein domains	-	-	-	-	-	-	-	-	-	-	-	+	-	-
scaffold516230-119582	kinetochore protein nuf2	-	-	-	-	-	-	-	-	+	-	-	-	-	-
scaffold538255.3-14297	ACT domain-containing protein	-	-	-	-	-	-	-	+	-	-	+	-	+	-
scaffold558498.1-17251	NA	-	+	-	-	-	-	-	-	-	-	-	-	-	-
scaffold562930-213796	uninformative,no protein domains	+	-	-	-	+	-	-	-	-	-	-	-	-	-
scaffold613301-392366	NA	-	-	-	-	-	-	-	-	-	-	-	+	-	-
scaffold63108.1-17228	zinc finger protein 6-like	+	+	-	-	+	+	+	-	-	-	-	-	-	-
scaffold66828.2-7495	Ovate protein family C-terminal	+	-	-	-	+	+	-	-	-	-	-	-	-	-
scaffold8376-82465	NA	-	-	-	-	-	-	-	-	-	-	-	+	-	-
scaffold847290-862	putative leucine-rich repeat receptor-like protein kinase, LRR_4	-	+	+	-	+	+	-	-	-	-	-	-	-	+
scaffold889382.1-27210	NA	-	-	-	-	-	-	-	+	-	-	-	-	-	-
scaffold898292-207672	protein transport protein Sec31A-like, WD40 repeat, Sec16_C	+	-	-	-	-	+	-	-	-	-	-	-	-	-
tscaffold1124-12427	NA	-	+	-	-	-	-	+	-	-	-	-	-	-	-
tscaffold1468-279791	NA	-	-	-	-	-	-	-	+	-	-	-	-	+	-
tscaffold1689-358859	NA	-	-	-	-	-	-	+	-	-	-	-	-	-	-
tscaffold2195-352205	ABC transporter B family member 26, chloroplastic	+	+	-	-	-	-	+	-	-	-	-	-	-	-
tscaffold2195-352260	ABC transporter B family member 26, chloroplastic	-	+	-	-	-	-	+	-	-	-	-	-	-	-
tscaffold2195-352404	ABC transporter B family member 26, chloroplastic	-	+	-	-	-	-	+	-	-	-	-	-	-	-
tscaffold2195-352405	ABC transporter B family member 26, chloroplastic	-	+	-	-	-	-	+	-	-	-	-	-	-	-
tscaffold239-3898685	NA	-	-	-	+	-	-	-	+	-	+	+	-	+	-
tscaffold2459-1053837	NA	-	-	-	-	-	-	-	-	-	-	-	+	-	-
tscaffold4288-27300	SANT/Myb domain,Signal transduction response regulator receiver domain,Myb_DNA-binding	+	-	-	-	-	-	-	-	-	-	-	-	-	-
tscaffold4288-27336	SANT/Myb domain,Signal transduction response regulator receiver domain,Myb_DNA-binding	+	+	-	-	-	-	-	+	-	-	-	-	-	-
tscaffold4288-27845	SANT/Myb domain,Signal transduction response regulator receiver domain,Myb_DNA-binding	+	-	-	-	-	-	-	-	-	-	-	-	-	-
tscaffold4288-27848	SANT/Myb domain,Signal transduction response regulator receiver domain,Myb_DNA-binding	+	-	-	-	-	-	-	-	-	-	-	-	-	-
tscaffold494-125995	NA	+	+	-	-	-	+	+	+	+	-	-	-	-	-
tscaffold5525-156106	UDP-glucuronosyl/UDP-glucosyltransferase	-	-	-	-	-	-	-	-	-	-	-	-	+	-
tscaffold5525-156312	UDP-glucuronosyl/UDP-glucosyltransferase	+	+	-	-	-	+	+	-	-	-	-	-	-	-
tscaffold5851-147146	NA	-	-	+	-	+	+	-	-	-	-	-	-	-	+
tscaffold6294-125489	RNA-binding KH domain-containing protein	+	-	-	-	-	+	-	-	-	-	-	-	-	-
tscaffold6897-130249	subtilisin-like protease-like, Peptidase S8/S53 domain	-	-	+	+	-	-	-	-	-	-	-	-	-	+
tscaffold6897-83128	subtilisin-like protease-like, Peptidase S8/S53 domain	-	-	-	-	-	-	+	-	-	-	-	-	-	-
tscaffold6952-66352	armadillo repeat-containing kinesin-like protein 2	-	+	-	-	-	+	+	-	-	-	-	-	-	-
tscaffold6952-66419	armadillo repeat-containing kinesin-like protein 2	-	+	-	-	-	+	+	-	-	-	-	-	-	-
tscaffold7760-235983	uninformative,no protein domains	+	-	-	-	-	-	-	-	-	-	-	-	-	-
tscaffold8039-203881	NA	-	-	-	-	-	-	+	+	+	-	-	-	-	-
tscaffold8439-118442	NA	-	-	-	-	-	-	-	-	-	-	+	-	-	-
tscaffold8439-118537	NA	+	-	-	-	-	-	-	-	-	-	+	-	-	-

Table S7 Background similarity tests for the south, north and west groups of *Pinus tabuliformis*.

Group for observed distribution	Group for background	Niche overlap		Observed SDM vs. expected SDM*
		Schoener's *D*	Warren's *I*	
North	South	0.03	0.14	Less
South	North	0.03	0.14	Less
North	West	0.11	0.30	Less
West	North	0.11	0.30	Less
South	West	0.03	0.10	Less
West	South	0.03	0.10	Less

Note: Niche similarity indexes of Schoener's *D* (Schoener 1968) and Warren's *I* (Warren et al. 2008) were examined.

*Background areas were defined by each species' distribution model (SDM) under the baseline threshold of minimizing the sum of sensitivity and specificity on the test data.

*$P < 0.001$ for *D* and *I*.
